# The prevalence of exercise-associated hyponatremia in 24-hour ultra-mountain bikers, 24-hour ultra-runners and multi-stage ultra-mountain bikers in the Czech Republic

**DOI:** 10.1186/1550-2783-11-3

**Published:** 2014-02-10

**Authors:** Daniela Chlíbková, Beat Knechtle, Thomas Rosemann, Alena Žákovská, Ivana Tomášková

**Affiliations:** 1 Centre of Sports Activities, Brno University of Technology, Brno, Czech Republic; 2 Institute of General Practise and for Health Services Research, University of Zurich, Zurich, Switzerland; 3 Institute of Experimental Biology, Faculty of Science, Masaryk University, Brno, Czech Republic; 4 Faculty of Forestry and Wood Sciences, Czech University of Life Sciences, Prague, Czech Republic; 5 Facharzt FMH für Allgemeinmedizin, Gesundheitszentrum St. Gallen, Vadianstrasse 26, 9001 St. Gallen, Switzerland

**Keywords:** 24-hour race, Multi-stage race, Fluid intake, Body mass, Sodium

## Abstract

**Background:**

To assess the prevalence of exercise-associated hyponatremia (EAH) in two 24-hour mountain bike (MTB) (R1,R2), one 24-hour running (R3) and one multi-stage MTB (R4) races held in the Czech Republic in a cluster of four cross-sectional studies.

**Methods:**

In 27 ultra-mountain bikers (ultra-MTBers), 12 ultra-runners, and 14 multi-stage MTBers, fluid intake, changes (Δ) in body mass, hematocrit, plasma volume, plasma [Na^+^], plasma [K^+^], plasma osmolality, urine [Na^+^], urine [K^+^], urine specific gravity, urine osmolality, K^+^/Na^+^ ratio in urine, transtubular potassium gradient and glomerular filtration rate were measured and calculated. The use of non-steroidal anti-inflammatory drugs and symptoms of EAH were recorded using post-race questionnaires.

**Results:**

Of the 53 finishers, three (5.7%) developed post-race EAH, thereof one (3.7%) ultra-MTBer, one (8.3%) ultra-runner and one (7.1%) multi-stage MTBer. Plasma [Na^+^] decreased significantly (*p* < 0.001) only in R4. Urine osmolality (R1, R3, R4 *p* < 0.001; R2 *p* < 0.05) and glomerular filtration rate (*p* < 0.001) increased, and body mass decreased in all races (*p* < 0.05). Δ body mass was inversely related to the number of kilometers achieved (*p* < 0.001) in R2 where better ultra-MTBers tended to lose more weight. Δ body mass (*p* < 0.001) and %Δ body mass (*p* = 0.05) were positively related to lower post-race plasma [Na^+^] in R3 that was associated with increased loss in body mass. Fluid intake was positively related to race performance in R1 and R2 (R1: *p* = 0.04; R2: *p* = 0.01) where ultra-MTBers in R1 and R2 who drank more finished ahead of those who drank less. Post-race plasma [Na^+^] was negatively associated with race performance in ultra-MTBers in R2 (*p* < 0.05), similarly ultra-runners in R3 (*p* < 0.05) where finishers with more kilometres had lower post-race plasma [Na^+^].

**Conclusions:**

The prevalence of EAH in the Czech Republic was no higher compared to existing reports on ultra-endurance athletes in other countries. Lower plasma [Na^+^] and development of EAH may be attributed to overdrinking, a pituitary secretion of vasopressin, an impaired mobilization of osmotically inactive sodium stores, and/or an inappropriate inactivation of osmotically active sodium.

## Background

Hydration status and its role in endurance performance is an important topic in exercise physiology. Recent studies investigated the changes in hydration status and the development of exercise-associated hyponatremia (EAH) in ultra-distance running races [1-15], in triathlon races [16-20], in mountain bike (MTB) multi-stage races [21-24], in single ultra-distance road cycling races [8,25,26], and in single ultra-distance MTB races [8,27,28]. However, 24-hour races have been investigated to a lesser extent [29-36]. Prior to 2010 there had been only one published study [9] investigating the prevalence of EAH in a single-stage ultra-marathon held in Europe.

Excessive fluid consumption leading to weight gain is thought to be the principal cause of reduced plasma [Na^+^] and previous studies in ultra-endurance events have shown an association between fluid intake, changes in body mass and plasma [Na^+^] [16,29,37-40]. However, in some studies a significant relationship between post-race plasma [Na^+^] and losses in body mass was reported [11,41]. EAH is most commonly found in athletes competing in ultra-endurance events and it is defined as plasma [Na^+^] < 135 mmol/l [39]. Signs and symptoms of EAH include nausea, vomiting, confusion, headache, seizures, pulmonary and cerebral oedema (hyponatremic encephalopathy), and possibly death [39]. Risk factors for EAH include low race pace, prolonged exercise with duration of more than four hours, female gender, a low body mass, pre-exercise hyperhydration, the use of non-steroidal anti-inflammatory drugs (NSAIDs), non-elite status, and extremely hot or cold environment [12,20,39,40].

Aside from the excessive fluid consumption associated with a high fluid availability and a sustained intake, EAH occurs due to an increased retention of fluid brought on by non-osmotic secretion of arginine vasopressin [12,42,43], elevated sweat sodium loss, the inability to mobilise exchangeable internal sodium stores, an inappropriate inactivation of osmotically-active sodium, metabolic water production, and an impaired renal blood flow or glomerular filtration [11,40].

Previous data on regular distance marathons have shown the prevalence of this fluid and electrolyte disorder to be at 22% [39]. However, in general, in ultra-endurance athletes, the prevalence of EAH should not exceed 10% [30], although there have been variable results in studies investigating the prevalence of EAH in ultra-marathons and other ultra-endurance events. Knechtle et al. reported that the prevalence of EAH was no higher in ultra-endurance athletes compared with existing reports on marathoners and Ironman triathletes [8], while studies of a single-stage mountain ultra-marathoner race [10], a 1,600-km multi-stage race [13] in Australia and a European 24-hour race showed no cases of EAH [10,30]. Conversely, in a recent study of 45 male ultra-marathoners in a 161-km ultra-marathon held in the USA, 51.2% of the finishers presented with EAH [7]. The longer nature of the 161-km ultra-marathon coupled with the prolonged period spent on the trail were assumed to be the main reasons for the increased prevalence of EAH, though when the data for five consecutive years were combined, the prevalence of EAH was shown to be 15.1% and positively related to ambient temperature [11]. In another study, 8% of mountain ultra-marathoners competing in a 7-stage race (350 km) in Switzerland developed EAH [8], while mild asymptomatic EAH was found to occur in 4% of the volunteer ultra-endurance mountain runners in New Zealand [9].

Studies investigating fluid intake and electrolyte metabolism balance have also been conducted in mountain ultra-endurance bike races. Studies of a single stage MTB race held in Switzerland [27,28] and multi-stage races in South Africa, the Alps (*i.e.* Germany, Austria, Switzerland and Italy) [21,22]. Similarly, no case of EAH was found in 65 ultra-endurance road cyclists competing in a 720-km ultra-cycling marathon in Switzerland [25]. On the contrary, 50% of the participants in an Alaskan cold weather race presented symptoms of EAH upon finishing the race [24].

Knechtle et al. described for 200 athletes competing in different disciplines in Switzerland that 12 finishers (6%) developed EAH [8]. The prevalence of EAH was 13% in swimmers, 10.7% for road cyclists, 8% for both ultra-marathoners and mountain ultra-marathoners and no case in mountain bikers. Regarding different disciplines, EAH was higher in running [1,3,4,6-12,38,39,44,45] compared to cycling [8,22,25,27,28]. However, according to recent findings the comparison of cyclists and runners is problematic because there are fewer studies of bike races [8].

There is a dearth of data on the prevalence of EAH in races held in Europe. Therefore, the aim of the current study was to investigate a series of ultra-endurance races held in the Czech Republic. Twenty-four-hour races held in different disciplines such as cycling and running are an ideal occasion to compare a prevalence of EAH between ultra-cyclists and ultra-runners. We intended to assess the prevalence of EAH in ultra-MTBers and ultra-runners in 24-hour races as single ultra-marathons and nearly non-stop performances without defined breaks with a specific load, and in a multi-stage race with an intermittent load with possibility of regeneration. We hypothesized an increased fluid intake during a 24-hour race for both cyclists and runners due to the large amount of fluids available at the refreshment stations in every lap. Since a slow running [31] or a slow cycling pace were considered as a main risk factor for fluid overload and development of EAH [21,25,29,38] we hypothesized that the prevalence of EAH would be higher in 24-hour races compared to a MTB stage race because there is a possibility of excessive drinking behavior and the duration of exercise is nearly 24 hours. Regarding recent studies, we hypothesized that the prevalence of EAH would be higher in runners compared to cyclists. Finally, it was hypothesized, that body mass loss in all races would have no influence on race performance [18,38,46,47]. In cases of fluid overload, we would expect post-race an increase in body mass [39] and a decrease in plasma [Na^+^] [12,39,48].

## Methods

### Ethics

Research within the project proceeded in accordance with the law (No. 96/2001 Coll. M. S. on Human Rights and Biomedicine and Act No. 101/2000 Coll. Privacy) and the study was approved by the local institutional ethics committee.

### Subjects (a cluster of four races)

Data were collected during four ultra-endurance races in the Czech Republic, were derived from four observational, cross-sectional studies and comprised athletes (*i.e.* ultra-MTBers, ultra-runners, and mountain bikers) participating in the, Czech Championship 24-hour MTB race‘ in Jihlava city (R1), in the‚ Bike Race Marathon Rohozec 24 hours‘ in Liberec city (R2), in the, Sri Chinmoy Self-Transcendence Marathon 24-hour race‘ in Kladno city (R3) and in the Trilogy Mountain Bike Stage Race‘ in Teplice nad Metují (R4) (see Tables 1 and 2).

**Table 1:** Description of races, Nr – number of race, TR – temperature range, AT – average temperature, AH – average relative humidity, weather, P – precipitation, F - finishers, prevalence of EAH (R1,R2,R3,R4)

**Nr**	**Type of race**	**TR (°C)**	**AT (°C)**	**AH (%)**	**Weather**	**P (mm)**	**F**	**Prevalence of EAH**
R1	24-h MTB race	6 – 30	18 (6)	43 (1)	Sun	—	12	0 (0%)
R2	24-h MTB race	6 – 23	15 (4)	72 (2)	Clouds	3 (2)	15	1 (6.7%)
R3	24-h running race	10 – 18	12 (3)	62 (3)	Rain	15(5)	12	1 (8.3%)
R4	Multi-stage race	22 – 33	26 (7)	55 (9)	Sun	—	14	1 (7.1%)

**Table 2:** Age, anthropometry, training, pre-race experience, and race performance of subjects (R1,R2,R3,R4), n = 53

	**Race 1 n = 12**	**Race 2 n = 15**	**Race 3 n = 12**	**Race 4 n = 14**
**Type of race**	**24-h MTB race**	**24-h MTB race**	**24-hour RUN race**	**Stage MTB race**
**Age, y**	40.3 (9.1)	36.8 (6.4)	38.3 (7.7)	38.0 (6.1)
**Body mass, kg**	75.2 (12.9)	72.1 (11.0)	66.3 (8.8)	75.3 (8.2)
**Body height, m**	178.1 (11.6)	176.7 (9.5)	174.8 (10.9)	176.6 (5.5)
**BMI, kg/m**^**2**^	23.5 (2.0)	23.0 (1.9)	21.7 (1.2)	24.1 (2.0)
**Years as active cyclist or runner**	10.3 (5.7)	8.6 (6.2)	9.8 (7.2)	11.4 (8.0)
**Number of finished ultra-marathons**	9.3 (7.2)	8.3 (7.3)	15.7 (19.3)	5.6 (6.6)
**Total training hours weekly, h**	12.3 (7.0)	12.1 (3.2)	10.6 (4.2)	10.7 (5.0)
**Training cycle or run hours weekly, h**	11.6 (6.2)	11.4 (3.2)	8.2 (3.4)	9.6 (3.9)
**Training intensity, b/min**	139.2 (6.7)	140.0 (9.3)	141.3 (18.8)	131.4 (12.3)
**Cases of EAH, absolute**	0	1	1	1
**Prevalence of EAH, %**	0	6.7	8.3	7.1

Results are presented as mean (SD). EAH = exercise-associated hyponatremia. R1: ,Czech Championship 24-hour MTB race‘ in Jihlava city, 24-hour MTB race, R2: Bike Race Marathon Rohozec 24 hours‘ in Liberec city, 24-hour MTB race, R3: Sri Chinmoy Self-transcendence Marathon 24-h race‘ in Kladno city, 24-hour RUN race, R4: Trilogy Mountain Bike Stage Race‘ in Teplice nad Metují, multi-stage MTB race (prolog: 3 km with 300 m difference in elevation, Stage 1: 66 km with 2,200 m, Stage 2: 63 km with 2,300 m and Stage 3: 78.8 km with 3,593 m).

Race participants were notified of the study approximately three months before the race start via an e-mail and were informed about the planned investigation with indication that participation was voluntary. Those who volunteered were instructed to keep a training diary until the start of the race. The training three months before the race, (*i.e.* number and duration of training units, training distance in kilometers and hours pre-race experience) were recorded.

A total of 58 athletes, thirteen recreational ultra-MTBers from 91 participants in solo category (R1), seventeen ultra-MTBers from 116 participants in solo category (R2), thirteen ultra-runners from 48 participants in solo category (R3) and fifteen MTBers from 206 participants (R4), all originating from the Czech Republic, agreed to participate (Table 2).

### Races (R1,R2,R3,R4)

The first measurement was performed at the, Czech Championship 24-hour MTB race‘ in Jihlava (R1), the race with the highest number of participants from the series of 24-hour MTB races held in the Czech Republic. The ultra-MTBers started at 12:00 on May 19^th^ 2012 and finished at 12:00 on May 20^th^ 2012. The course was comprised of a 9.5 km single-track with an elevation of 220 m. A single aid station, located at the start/finish area was provided by the organizer where a variety of food and beverages such as hypotonic sports drinks, tea, soup, caffenaited drinks, water, fruit, vegetables, energy bars, bread, soup, sausages, cheese, bread, chocolate and biscuits were available. The ultra-MTBers could also use their own supplies in their pitstops. The maximum temperature was +30°C, the minimum temperature was +6°C during the night on some places of the route and the average temperature was +18 (6)°C. No precipitation was recorded and relative humidity was at 43 (12)% over the duration of the race.

The largest and the oldest (18^th^ edition) 24-hour cycling race in the Czech Republic with the longest tradition, the‚ Bike Race Marathon Rohozec‘ in Liberec (R2), took place from June 9^th^ 2012 to June 10^th^ 2012. The course was comprised of a 12.6 km track with an elevation of 250 m. The track surface consisted of paved and unpaved roads and paths. There was one aid station located at the start and finish with food and beverages similar to those mentioned above. The maximum temperature was +23°C, the minimum temperature was +6°C during the night and the average temperature was +15 (4)°C. Over the duration of the race, 3 (1.5) mm of precipitation was recorded and relative humidity varied from 44% till 98%.

The, Sri Chinmoy Self-transcendence Running Marathon 24- hour race‘ (R3) took place from July 21^st^ 2012 to July 22^nd^ 2012 in Kladno. We chose this race because it was the largest 24-hour running race in the Czech Republic with the highest number of participants and we also wanted to compare ultra-runners with ultra-MTBers. The lap was 1 km, situated around an athletic stadium on asphalt with 1 m rise. The athletes could consume food and beverages *ad libitum* from a buffet provided by the organizer with warm and cool food like apples, ananas, oranges, dried fruit, potatoes, rice, cookies, bread, pasta, porridge, soup, water, tea, isotonic drinks, fruit juices, cola, broth, and coffee. Runners could place their own camping tables and chairs with personal belongings, food and drinks in a designated area. The maximum temperature was +18°C, the minimum temperature was +10°C, and the average temperature was +12 (3)°C. On average 15 (5) mm of precipitation was recorded and relative humidity changed from 58 till 94% over the duration of the race.

The ,Trilogy Mountain Bike Stage Race‘ (R4), the first MTB stage race held in the Czech Republic, took place from July 4^th^ 2012 till July 8^th^ 2012 in Teplice nad Metují. This four day race consisted of a prologue and three stages, each of which had a completely different character. We chose this race to compare 24-hour races with a stage race. The difficulty of this race was similar to other stage MTB races in Europe. The prologue covered 3 km with 300 m difference in elevation, Stage 1 covered 66 km with 2,200 m of altitude to climb, Stage 2 was 63 km in length with 2,300 m difference in elevation and Stage 3 was 78.8 km with 3,593 m. Stage routes were characterized by a large number of individual trails which only interrupted by a necessary minimum of road sections. Aid stations located along the routes offered beverages such as hypotonic sports drinks, tea, soup, caffenaited drinks, water, fruit, vegetables, energy bars, bread, soup, sausages, cheese, bread, chocolate and biscuits. During the prologue the average temperature was +32 (1)°C and relative humidity was 55 (2)% over the duration of the race. At Stage 1 the maximum temperature was +33°C, the minimum +22°C, the average temperature was +27 (7)°C and relative humidity changed from 80% at the start till 48% at the end of the race. At Stage 2 the maximum temperature was +30°C, the minimum +22°C, the average temperature +25 (3)°C and relative humidity changed from 83% till 60% over the duration of the race. At Stage 3 the maximum temperature was +31°C, the minimum +19°C, the average temperature was +25 (2)°C and relative humidity changed from 85% till 37% over the duration of the race.

### Procedures and calculations

The procedures of pre- and post-race measurements were identical. At first, pre-race anthropometry of the subjects was assessed. Athletes were measured after voiding their urinary bladder. Body mass was measured using a calibrated commercial scale (Tanita BC-351, Tanita corporation of America, Inc.) to the nearest 0.1 kg. Subjects were barefoot and generally clothed in cycling attire for both the pre- and post-race measurements. Body height was determined using a stadiometer (Harpenden Stadiometer, Baty International Ltd) to the nearest 0.01 m. Body mass index was calculated using body mass and body height. Blood samples were drawn from an antecubital vein. Standardization of the sitting position prior to blood collection was respected since postural changes can influence blood volume and concentration of hematocrit. One Sarstedt S-Monovette (plasma gel, 7.5 ml) for chemical and one Sarstedt S-Monovette (EDTA, 2.7 ml) for hematological analysis were cooled and sent to the laboratory and were analysed within 6 hours. Blood samples were obtained to determine pre- and post-race hematocrit, plasma [Na^+^], plasma [K^+^], and plasma osmolality. Hematocrit was determined using Sysmex XE 2100 (Sysmex Corporation, Japan), plasma [Na^+^] and plasma [K^+^] were determined using biochemical analyzer Modula SWA, Modul P + ISE (Hitachi High Technologies Corporation, Japan, Roche Diagnostic), and plasma osmolality was determined using Arkray Osmotation (Arkray Factory, Inc., Japan). Samples of urine were collected in one Sarstedt monovett for urine (10 ml) and sent to the laboratory. In urine samples, pre- and post-race [Na^+^], [K^+^], specific gravity and osmolality were determined. Urine [Na^+^], urine [K^+^] and urine urea were determined using biochemical analyzer Modula SWA, Modul P + ISE (Hitachi High Technologies Corporation, Japan, Roche Diagnostic), urine specific gravity was determined using Au Max-4030 (Arkray Factory, Inc., Japan), and osmolality was determined using Arkray Osmotation (Arkray Factory, Inc., Japan). Transtubular potassium gradient was calculated using the formula (potassium_urine_ × osmolality_serum_)/(potassium_serum_ × osmolality_urine_) [49]. Glomerular filtration rate was calculated using the formula of Levey et al. [50]. K^+^/Na^+^ ratio in urine was calculated. Percentage change in plasma volume was calculated from pre- and post-race values of hematocrit using the equation of van Beaumont [51]. In an effort to maintain impartial interpretation, the results were not reviewed at the time and no opportunity existed to recommend for or against participation in the races.

Pre-race testing took place during the event’s registration in the morning before the race between 07:00 a.m. and 11:00 a.m. in the morning in 24-hour races and three hours before the start of the prolog in the multi-stage race. The athletes were informed of the procedures and gave their informed written consent. No measurements were taken during the race. During the race fluid consumption was recorded by the athlete or by one of the support team on a recording sheet. At each aid station, they marked the number of cups of fluid consumed. In addition, all fluid intake provided by the support crew was recorded. The athletes primarily ate and drank as they ran or cycled. Fluid intake was estimated according to the reports of the athletes. The organizer provided no special advice on the web site about what and how much the athletes should drink during the race. Post-race measurements were taken immediately after the races and were finished within two hours in the 24-hour races, when all finishers ended the race and some of them were finally able to hand in urine samples due to problems with antidiuresis. Post-race measurements were taken directly after arrival at the finish line after every stage in the multi-stage MTB race. Questionnaires were also issued at these times and athletes gave information of the use of non-steroidal anti-inflammatory drugs [9] during the races and symptoms of EAH [12,40].

### Statistical analysis

Results are presented as mean and standard deviation (SD) as appropriate. The Shapiro-Wilk test was applied to check for normal distribution of data. Paired sample *t*-tests or the Wilcoxon signed-rank tests were used to compare laboratory parameters before and after the race as appropriate and to compare continuous measures. The results were compared using the Mann-Whitney *U -*test. The correlations of the changes in parameters during the race were evaluated using Pearson product-moment to assess the univariate associations. For all statistical tests, significance was set at a level of 0.05.

## Results

Out of the 58 athletes recruited, 53 (91.4%), such as 12 ultra-MTBers (R1), 15 ultra-MTBers (R2), 12 ultra-runners (R3), and 14 MTBers (R4) successfully completed one of the four races (R1-R4) and passed both pre- and post-race measurements (Table 1). One cyclist had to give up due to an equipment failure, while four others had to quit the race because of medical complications or physical exhaustion. Hyponatremia was not evident in those who failed to complete their respective race. Table 2 summarizes anthropometric and training characteristics of the 50 finishers without EAH from all races (R1-R4).

### Prevalence of exercise-associated hyponatremia

In three subjects (EAH-A-R2, EAH-B-R3, EAH-C-R4), post-race plasma [Na^+^] varied between 129 and 134 mmol/l corresponding biochemically to a mild to medium hyponatremia (Table 3). Based on the classification by Noakes et al. [39], hypernatremia is defined as a serum [Na^+^] ≥ 145 mmol/l, normonatremia as a serum [Na^+^] = 135 - 144.9 mmol/l, biochemical hyponatremia as a serum [Na^+^] = 129 - 134.9 mmol/l, and clinical hyponatremia as a serum [Na^+^] ≤ 129 mmol/l. The prevalence of post-race EAH in 24-hour MTB races (R1,R2) in the Czech Republic was 3.7% from 27 ultra-MTBers. No ultra-MTBer in R1 developed post-race EAH. One ultra-MTBer in R2 (EAH-A-R2) showed post-race EAH, where plasma [Na^+^] dropped from 138 mmol/l pre-race to 129 mmol/l post-race. Two other ultra-MTBers - one each from R1 and R2 - started their races with plasma [Na^+^] indicating hyponatremia (132 mmol/l), however they developed no post-race hyponatremia. No ultra-MTBer (R1,R2) showed pre-race or post-race hypernatremia. The estimated prevalence of EAH in the 24-hour running race (R3) was 8.3% from 12 ultra-runners. One ultra-runner (EAH-B-R3) developed EAH, as his plasma [Na^+^] dropped from 137 mmol/l pre-race to 133 mmol/l post-race. No ultra-runner showed pre-race or post-race hypernatremia. The estimated prevalence of EAH in the multi-stage MTB race was 7.1% from 14 MTBers. One MTBer (EAH-C-R4) developed EAH, as his plasma [Na^+^] dropped from 142 mmol/l pre-race to 134 mmol/l post-race. No MTBer developed pre-race or post-race hypernatremia.

**Table 3:** Characteristics of the three cases (EAH-A-R2, EAH-B-R3, EAH-C-R4) with exercise-associated hyponatremia (n = 3)

	**EAH-A-R2**	**EAH-B-R3**	**EAH-C-R4**
**Type of race**	**24-h MTB race**	**24-h RUN race**	**Multi-stage MTB race**
**Age (years)**	39	38	42
**Body height (m)**	196	168	177
**BMI (kg/m**^**2**^**)**	23.4	18.8	23.6
**Pre-race body mass (kg)**	90.0	54.6	73.9
**Post-race body mass (kg)**	88.2	53.2	71.7
**Δ body mass (kg)**	–1.8	–1.4	–2.2
**Δ body mass (%)**	–2.0	–2.6	–3.0
**Pre-race plasma sodium (mmol/l)**	138.0	137.0	142.0
**Post-race plasma sodium (mmol/l)**	129.0	133.0	134.0
**Δ haematocrit (%)**	–7.6	–9.4	3.8
**Δ plasma potassium (mmol/l)**	32.6	–29.2	3.6
**Δ plasma osmolality (mosmol/kg H**_**2**_**O)**	–0.7	–1.1	1.7
**Pre-race urine specific gravity (g/ml)**	1.015	1.010	1.007
**Post-race urine specific gravity (g/ml)**	1.025	1.025	1.028
**Δ urine osmolality (mosmol/kg H**_**2**_**O)**	338:9	163.5	228.0
**Δ urine potassium (mmol/l)**	323.2	90.5	1282.0
**Δ urine sodium (mmol/l)**	108.3	25.9	–71.4
**Δ K/Na in urine (%)**	103.1	51.2	4737.0
**Δ Transtubular potassium gradient (%)**	1262.5	611.4	4340.0
**Years as active cyclist or runner**	5	15	5
**Number of finished ultra-marathons**	4	30	2
**Total training hours weekly, h**	12	13	10
**Training cycle or run hours weekly, h**	10	30	10
**Training intensity, b/min**	140	130	140

The intake of NSAIDs was reported by 3 (25%) of 12 ultra-runners and by no cyclist (from 41) in any race. Regarding symptoms associated with race performance in R1, most of ultra-MTBers without EAH noted in the post-race questionnaires muscle weakness (41.7%), problems with antidiuresis (33.3%), and breathing problems (33.3%). Muscle weakness (46.7%), problems with antidiuresis (40%), headache (26.7%), and breathing problems (26.7%) were the most reported post-race symptoms associated with race performance in R2 by finishers without EAH. Chills (50.8%), stomach pain (33.3%) and irritability (33.3%) were the most noted post-race symptoms associated with race performance in R3 by ultra-runners without EAH. MTBers without EAH reported muscle weakness (50%), swelling (42.9%) and myalgia (35.7%) in R4.

Subjects who exhibited hyponatremia reported no intake of NSAIDs during the study period. The ultra-MTBer (EAH-A-R2) reported muscle weakness. The ultra-runner (EAH-B-R3) in a 24-hour running race R3 with EAH reported symptoms like antidiuresis, headache, flushing, irritability, dizziness, myalgia, disorientation, lethargy, swelling and mental status change. The MTBer (EAH-C-R4) in the multi-stage MTB race (R4) with EAH reported symptoms like antidiuresis, muscle weakness, myalgia and swelling. EAH-A-R2, EAH-B-R3 and EAH-C-R4 developed a post-race biochemical EAH.

### Hyponatremic finishers (n = 3) and their anthropometric parameters, parameters of hydration status, and fluid intake

Anthropometric parameters, blood and urine parameters, pre-race training logs of hyponatremic cases EAH-A-R2, EAH-B-R3 and EAH-C-R4 are summarized in Table 3. Pre and post-race results for physical, blood and urine parameters are shown in Table 4. For comparison purposes, we used cut-off points for hydration state based upon Δ body mass by Noakes et al. [39], where ≥ 0 Δ body mass is overhydration, < 0 to -3% Δ body mass is euhydration, and < -3% Δ body mass is dehydration. A decrease was seen in both body mass and Δ body mass, respectively, in EAH-A-R2 (1.8 kg, 2.0%) and EAH-B-R3 (1.4 kg, 2.6%). In EAH-C-R4, decreases in body mass (2.2, 2.8, 2.2 kg) and Δ body mass (3.0%, 3.8%, 3.0%) were seen after Stage 1, 2 and 3 respectively. EAH-A-R2 consumed 0.90 l/h, EAH-B-R3 and EAH-C-R4 each consumed 0.75 l/h, which equated to 0.010 l/kg in EAH-A-R2, 0.014 l/kg in EAH-B-R3, 0.010 l/kg in EAH-C-R4; which was not related to race speed, ambient temperature or relative humidity during the race (*p* > 0.05).

**Table 4:** Physical, blood and urine parameters before and after the race (n = 3)

	**Pre-race**	**Post-race**	**Change (absolute)**	**Change (%)**
Body mass (kg)	72.8 (12.5)	71.0 (12.4)	–1.8 (0.4)*	–2.5 (0.5)*
Haematocrit (%)	42.7 (1.1)	40.9 (3.2)	–1.8 (3.1)	–4.4 (7.2)
Plasma sodium (mmol/l)	139 (1.9)	132 (1.9)	–7 (2.6)*	–5 (1.9)*
Plasma potassium (mmol/l)	5.5 (0.7)	5.5 (0.5)	–0.1 (1.7)	–2.3 (30.9)
Plasma osmolality (mosmol/kg H_2_O)	287.7 (3.6)	287.7 (5.5)	0.0 (4.4)	0.0 (1.5)
Urine specific gravity (g/ml)	1.011 (0.003)	1.026 (0.001)	0.020 (0.010)*	1.520 (0.550)*
Urine osmolality (mosmol/kg H_2_O)	204.0 (36.9)	681.0 (97.2)	477.0 (132.9)*	243.5 (88.7)*
Urine potassium (mmol/l)	17.2 (8.1)	81.2 (35.1)	64.0 (55.8)	565.2 (631.6)
Urine sodium (mmol/l)	40.0 (10.7)	43.3 (20.2)	3.3 (29.5)	20.9 (90.0)
K/Na ratio in urine	0.4 (0.1)	4.4 (0.1)	4.0 (6.3)	1630.5 (2690.5)
Transtubular potassium gradient	2.3 (1.3)	32.5 (8.0)	30.2 (11.9)*	2071.3 (1991.6)*
Glomerular filtration rate (ml/min)	91.9 (6.6)	64.2 (13.3)	–27.8 (28.1)	–28.5 (26.1)

Results are presented as mean (SD), *= p ≤ 0.05.

### Normonatremic finishers (n = 50) and their anthropometric parameters, parameters of hydration status, and fluid intake

#### Race 1 - R1 (24-hour MTB race)

**Figure 1:**
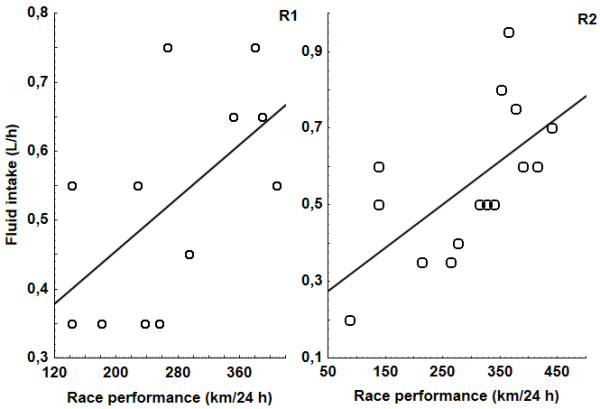
**Fluid intake was positively related to race performance (R1: *r*** **= 0.58, *p*** **= 0.05; R2: *r*** **= 0.63, *p*** **= 0.01).** R1: Czech Championship 24-hour MTB race‘ in Jihlava city, 24-hour MTB race, R2: ,Bike Race Marathon Rohozec 24 hours‘ in Liberec city, 24-hour MTB race.

For all finishers body mass significantly decreased (*p* < 0.001) in R1, Δ body mass was -2.0 kg (2.7%). In the one (8.3%) ultra-MTBer, body mass increased by 0.1 kg. In the remaining 11 cyclists, body mass decreased between 1.0 kg and 5.1 kg. Three of them (25.0%) were dehydrated according to Noakes et al. [39]. The Δ body mass or % Δ body mass were neither related to plasma [NA^+^], Δ plasma [Na^+^] or race performance. Finishers showed a significantly increase in urine specific gravity (*p* < 0.001). Increased post-race urine osmolality (*p* < 0.001) was significantly related to increased post-race urine [K^+^] (*p* < 0.001) (r = 0.61, *p* < 0.05). K^+^/Na^+^ ratio in urine significantly increased (*p* < 0.001) and it was > 1. Plasma volume decreased by 3.9% (12.6%); Δ plasma volume was not related to post-race plasma osmolality, or to post-race urine osmolality. Hematocrit, plasma [Na^+^], plasma [K^+^], plasma osmolality, and urine [Na^+^] remained stable (see Table 5). Post-race plasma [Na^+^] was significantly and positively related to Δ plasma [Na^+^] (*r* = 0.78, *p* < 0.001). Transtubular potassium gradient significantly increased (*p* < 0.001), and glomerular filtration race significantly decreased (*p* < 0.001). Ultra-MTBers consumed a total of 0.49 (0.15) l/h during the 24-hour MTB race. Fluid intake varied between 0.30 l/h and 0.70 l/h and was positively related to the number of achieved kilometers (race performance) during the 24-hour MTB race (*r* = 0.58, *p* = 0.04) (Figure 1). Fluid intake showed no correlation to body mass, Δ body mass, post-race plasma [Na^+^], Δ plasma [Na^+^], Δ plasma volume or USG.

**Table 5:** (A,B,C,D) - Changes in blood and urine parameters (R1,R2,R3,R4) in subjects without EAH, n = 50

**A**	**Pre-race**			
**Parameter**	**R1**	**R2**	**R3**	**R4**
**Haematocrit (%)**	41.7 (3.7)	41.8 (3.0)	42.1 (3.2)	41.7 (2.3)
**Plasma sodium (mmol/l)**	138.0 (2.7)	137.7 (2.1)	140.0 (1.7)	141.8 (1.9)
**Plasma potassium (mmol/l)**	6.5 (1.5)	4.6 (0.3)	6.6 (0.9)	5.1 (0.4)
**Plasma osmolality (mosmol/kg H**_**2**_**O)**	289.9 (5.0)	289.4 (4.7)	288.6 (3.4)	288.7 (3.4)
**Urine specific gravity (g/ml)**	1.015 (0.004)	1.016 (0.004)	1.013 (0.005)	1.015 (0.007)
**Urine osmolality (mosmol/kg H**_**2**_**O)**	485.01 (219.1)	530.01 (272.3)	364.8 (163.3)	444.4 (273.0)
**Urine potassium (mmol/l)**	28.3 (28.9)	50.4 (37.7)	28.3 (15.8)	37.0 (28.9)
**Urine sodium (mmol/l)**	58.7 (46.1)	82.8 (40.8)	81.3 (39.5)	94.2 (52.3)
**K/Na ratio in urine**	0.5 (0.4)	0.6 (0.4)	0.4 (0.2)	0.5 (0.4)
**Transtubular potassium gradient**	6.9 (6.7)	25.7 (28.9)	7.0 (7.0)	15.5 (22.1)
**Glomerular filtration rate (ml/min)**	86.9 (15.0)	82.9 (8.6)	93.0 (7.6)	86.9 (8.2)
**B**	**Post-race**			
**Parameter**	**R1**	**R2**	**R3**	**R4**
**Haematocrit (%)**	42.8 (3.0)	40.8 (2.8)	40.8 (2.9)	39.7 (2.9)
**Plasma sodium (mmol/l)**	137.4 (2.6)	136.8 (2.8)	138.7 (2.5)	139.2 (2.5)
**Plasma potassium (mmol/l)**	6.1 (1.0)	4.6 (0.9)	5.0 (0.6)	5.1 (0.5)
**Plasma osmolality (mosmol/kg H**_**2**_**O)**	292.7 (4.2)	291.8 (5.0)	290.4 (6.0)	290.1 (4.4)
**Urine specific gravity (g/ml)**	1.021 (0.004)	1.022 (0.004)	1.019 (0.010)	1.025 (0.007)
**Urine osmolality (mosmol/kg H**_**2**_**O)**	764.3 (196.9)	730.9 (241.4)	505.0 (312.0)	763.4 (291.4)
**Urine potassium (mmol/l)**	77.8 (25.4)	61.9 (47.9)	44.2 (27.8)	76.3 (31.2)
**Urine sodium (mmol/l)**	43.2 (30.6)	44.4 (44.9)	51.2 (34.7)	80.4 (58.9)
**K/Na ratio in urine**	2.3 (1.0)	2.3 (2.7)	0.9 (0.6)	2.2 (3.0)
**Transtubular potassium gradient**	35.6 (19.7)	40.3 (41.4)	20.5 (17.7)	42.8 (22.6)
**Glomerular filtration rate (ml/min)**	69.6 (12.4)	71.2 (9.9)	86.2 (9.5)	72.3 (12.2)
**C**	**Change (absolute)**			
**Parameter**	**R1**	**R2**	**R3**	**R4**
**Haematocrit (%)**	1.1 (3.2)	–1.0 (2.6)	–1.3 (2.0)*	–2.0 (2.2)
**Plasma sodium (mmol/l)**	–0.6 (3.9)	–0.9 (2.8)	–1.3 (2.6)	–2.6 (2.4)**
**Plasma potassium (mmol/l)**	–0.4 (1.7)	–0.1 (1.0)	–1.6 (1.1)**	–0.0 (0.5)
**Plasma osmolality (mosmol/kg H**_**2**_**O)**	2.8 (4.2)	2.4 (6.2)	1.8 (5.8)	1.4 (5.5)
**Urine specific gravity (g/ml)**	0.006 (0.004)**	0.006 (0.006)**	0.006 (0.011)	0.010 (0.009)**
**Urine osmolality (mosmol/kg H**_**2**_**O)**	279.3 (195.1)**	200.8 (342.4)*	114.2 (355.8)**	319.0 (326.9)**
**Urine potassium (mmol/l)**	49.5 (39.0)**	11.5 (22.9)	15.9 (35.9)	39.3 (40.6)**
**Urine sodium (mmol/l)**	–15.6 (37.5)	–37.8 (46.0)**	–30.1 (38.4)*	–13.8 (70.3)
**K/Na ratio in urine**	1.7 (0.7)**	1.7 (2.4)*	0.56 (0.7)*	1.7 (3.1)
**Transtubular potassium gradient**	28.7 (14.1)**	14.6 (46.4)	13.5 (20.0)*	27.3 (23.7)**
**Glomerular filtration rate (ml/min)**	–17.2 (14.7)**	–11.7 (9.8)**	–6.8 (6.1)**	–14.6 (14.2)**
**D**	**Change (%)**			
**Parameter**	**R1**	**R2**	**R3**	**R4**
**Haematocrit (%)**	2.8 (8.0)	–2.4 (6.1)	–3.1 (4.8)*	–4.8 (5.1)
**Plasma sodium (mmol/l)**	–0.4 (2.8)	–0.6 (2.0)	–0.9 (1.9)	–1.8 (1.7)**
**Plasma potassium (mmol/l)**	–6.3 (27.8)	–0.6 (22.9)	–24.6 (14.1)**	–1.0 (9.2)
**Plasma osmolality (mosmol/kg H**_**2**_**O)**	0.9 (1.5)	0.8 (2.1)	0.6 (2.0)	0.5 (1.9)
**Urine specific gravity (g/ml)**	0.7 (0.4)**	0.5 (0.6)**	0.6 (1.1)	1.0 (0.9)**
**Urine osmolality (mosmol/kg H**_**2**_**O)**	57.6 (85.3)**	37.9 (161.2)*	38.4 (195.6)**	71.8 (185.1)**
**Urine potassium (mmol/l)**	174.8 (458.2)**	22.9 (22.0)	56.3 (191.8)	106.2 (422.9)**
**Urine sodium (mmol/l)**	–26.5 (81.6)	–46.0 (118.1)**	–37.0 (36.1)*	–14.6 (105.2)
**K/Na ratio in urine**	359.9 (187.2)**	281.7 (327.6)*	148.7 (222.3)*	366.6 (347.9)
**Transtubular potassium gradient**	417.7 (575.1)**	56.9 (1185.7)	192.7 (2133.5)*	176.6 (1196.2)**
**Glomerular filtration rate (ml/min)**	–19.8 (14.7)**	–14.1 (11.7)**	–7.3 (6.7)**	–16.8 (13.9)**

Results are presented as mean (SD), *= p ≤ 0.05, **= p < 0.01.

#### Race 2 - R2 (24-hour MTB race)

BM decreased (*p* < 0.05), Δ BM was -1.3 kg (1.9%), finishers were euhydrated following the definition of Noakes et al. [39]. In four (26.7%) ultra-MTBers, body mass increased between 0.2 kg and 2.0 kg. In the remaining 11 ultra-MTBers, body mass decreased between 0.7 kg and 5.0 kg, indicating that five (33.3%) were dehydrated. Δ body mass was negatively and significantly related to race performance (*r* = -0.69, *p* < 0.001). Neither Δ body mass nor% Δ body mass were related to post-race plasma [Na^+^] or Δ plasma [Na^+^]. Hematocrit, plasma [Na^+^], plasma [K^+^], plasma osmolality, and transtubular potassium gradient remained stable (see Table 5). Post-race plasma [Na^+^] was significantly and positively related to Δ plasma [Na^+^] (*r* = 0.72, *p* < 0.001). Plasma volume increased by 4.9% (11.3%) and Δ plasma volume was not related to an increased K^+^/Na^+^ ratio in urine > 1 (*p* < 0.05), plasma osmolality, or to urine osmolality. Urine [Na^+^] and glomerular filtration race significantly decreased (*p* < 0.001), and urine specific gravity increased (*p* < 0.001). Ultra-MTBers consumed a total of 0.55 (0.19) l/h during the 24-hour MTB race. Fluid intake varied between 0.20 l/h and 0.95 l/h and correlated significantly and positively to race performance (*r* = 0.62, *p* = 0.01) (Figure 1) as in ultra-MTBers in R1. Fluid intake showed no correlation to post-race body mass, Δ body mass, Δ plasma volume or urine specific gravity. However, post-race plasma [Na^+^] was negatively associated with race performance in ultra-MTBers (*r* = -0.53, *p* < 0.05) (Figure 1), as in ultra-runners in R3. Also, fluid intake was negatively related to post-race plasma [Na^+^] (*r* = 0.56, *p* < 0.05).

#### Race 3 - R3 (24-hour running race)

Body mass decreased (*p* < 0.05), Δ body mass was -0.9 kg (1.4%). In two (16.7%) ultra-runners body mass increased by 0.1 kg and 1.0 kg, indicating overhydration according to Noakes et al. [39]. In the remaining 10 ultra-runners, body mass decreased between 0.3 kg and 2.7 kg, two (16.7%) ultra-runners were dehydrated. Δ body mass was neither related to Δ plasma [Na^+^] nor race performance. Δ body mass (*r* = 0.78, *p* < 0.001), and% body mass (*r* = 0.58, *p* = 0.05) were positively related to post-race plasma [Na^+^]. Race performance was negatively associated with post-race plasma [Na^+^] (*r* = -0.64, *p* < 0.05), similarly as in R2. Post-race plasma [Na^+^] was significantly and positively related to Δ plasma [Na^+^] (*r* = 0.78, *p* < 0.001). Plasma volume increased by 5.9% (8.7%) (*p* < 0.05), while Δ plasma volume was not related to post-race plasma osmolality, or to post-race urine osmolality. Hematocrit and urine [Na^+^] (*p* < 0.05), glomerular filtration race and plasma [K^+^] (*p* < 0.001) significantly decreased. K^+^/Na^+^ ratio in urine increased (*p* < 0.05), but was < 1. In contrast, urine osmolality increased significantly (*p* < 0.001) (Table 5). Ultra-runners consumed a total of 0.58 (0.38) l/h during a 24-hour running race. Fluid intake varied between 0.15-0.90 l/h and showed no association with race performance, body mass, Δ body mass, post-race plasma [Na^+^], Δ plasma [Na^+^], Δ plasma volume or post-race urine specific gravity.

#### Race 4 - R4 (multi-stage MTB race)

Body mass decreased (*p* < 0.05), Δ body mass was -1.6 kg (2.1%) after Stage 1 (*p* < 0.001); -1.7 kg (-2.3%) after Stage 2 (*p* < 0.001), and -1.3 kg (-1.7%) after Stage 3 (*p* < 0.05). Body mass decreased in all MTBers after Stage 1, three (21.4%) were dehydrated according to Noakes et al. [39]. In one (7.9%) MTBer, body mass remained stable, in another (7.9%) MTBer body mass increased by 0.1 kg, indicating overhydration, while in the remaining 12 MTBers body mass decreased between 0.3 kg and 3.9 kg, five (35.7%) MTBers were dehydrated after Stage 2. In three (21.4%) MTBers, body mass increased between 0.5 kg and 2.4 kg, they were overhydrated, and in the remaining 11 MTBers body mass decreased between 0.6 kg and 3.4 kg, six (42.9%) MTBers were dehydrated after Stage 3. Δ body mass or % Δ body mass were neither related to Δ plasma [Na^+^], post-race plasma [Na^+^], nor race performance. Plasma [Na^+^], and glomerular filtration race decreased significantly (*p* < 0.001), and plasma volume increased by 5.3% (5.7%), Δ plasma volume was not related to post-race plasma osmolality, or to post-race urine osmolality. Post-race plasma [Na^+^] was significantly and positively related to Δ plasma [Na^+^] (*r* = 0.71, *p* < 0.001). In contrast, urine specific gravity, urine osmolality and urine [K^+^] increased significantly (*p* < 0.001), K^+^/Na^+^ ratio in urine did not increase significantly and was > 1 post-race. Urine specific gravity was associated with urine [K^+^] (*r* = 0.70, *p* < 0.001). Transtubular potassium gradient increased significantly (*p* < 0.001) (Table 5). Multi-stage ultra-MTBers consumed approximately a total of 0.43 (0.3) l/h during every stage. Fluid intake varied between 0.2-0.85 l/h and showed no association with achieved race time from all stages. Fluid intake showed no correlation to post-race body mass, Δ body mass, post-race plasma [Na^+^], Δ plasma [Na^+^], Δ plasma volume or Δ urine specific gravity.

## Discussion

The aim of the study was to investigate the prevalence of EAH in ultra-endurance athletes such as ultra-MTBers, ultra-runners and MTBers in four races held in the Czech Republic, Europe. The most important finding was that three (5.7%) of the 53 finishers developed post-race EAH with post-race plasma [Na^+^] < 135 mmol/l. The prevalence of EAH in the Czech Republic was not higher than in other reports from Europe. Moreover, symptoms typical of EAH were also reported in normonatremic competitors.

### Prevalence of EAH in all races (R1,R2,R3,R4)

The prevalence of post-race EAH varied from 0% to 8.3% in the individual races. No ultra-MTBer developed EAH in the 24-hour MTB race R1. One ultra-MTBer in the 24-hour MTB race (R2), one ultra-runner in the 24-hour running race (R3) and one MTBer in the multi-stage MTB race (R4) showed EAH with mild clinical symptoms. Furthermore, two (3.7%) athletes (R2) presented with pre-race EAH, and no finisher was pre- or post race hypernatremic. The work herein failed to support the hypothesis that the prevalence of EAH would be higher in 24-hour races compared with the multi-stage MTB race. The prevalence of EAH in all 24-hour races (R1,R2,R3) was 5.4% for 39 athletes and 7.1% for 14 athletes in the multi-stage MTB race (R4). The prevalence of EAH was lower in ultra-MTBers compared to ultra-runners and MTBers. The current work also demonstrated that the prevalence of EAH was higher in ultra-runners compared to ultra-MTBers. In contrast with the results of the current study, EAH occurred in more than 50% of the finishers of a 161-km ultramarathon in California which took place on single track mountain trails similar those in R1 and R2 in the present study [7]. Studies on EAH in ultra-running events in Switzerland [30] and New Zealand [9] reported prevalences of 0% and 4% respectively compared to the 8.3% reported here. The prevalence of EAH in ultra-MTBers (3.7%) and MTBers (7.1%) in the current study was also similar to studies of multi-stage MTB races in South Africa and the Alps [21,22], as well as single ultra-distance road cycling and MTB races in Switzerland [8,25-28]. On average, post-race EAH in the Czech Republic amounted to 5.7% and did not exceed 10%. Regarding existing reports on EAH in single ultra-distance running races [1,3,4,6-12,38,39], in MTB multi-stage races [21,22], in single ultra-distance MTB races [8,22,25,28] the prevalence rates in the Czech Republic were no higher in the present athletes.

An interesting finding was that the normonatremic group reported also symptoms typical for EAH. Muscle weakness, antidiuresis and breathing problems were the most reported post-race symptoms in finishers in the 24-hour cycling races (R1, R2). Moreover, swelling and myalgia occurred in the multi-stage race alongside reported muscle weakness. The presented problems with antidiuresis could be associated with dehydration and SIADH (syndrome of inappropriate secretion of antidiuretic hormone). On the contrary, symptoms like chills, stomach pain and irritability in runners (R3) were probably more associated with race performance and were influenced by weather conditions. Post-race, all finishers, both hyponatremic and normonatremic, presented without symptoms of altered mental status. No subject required medical attention for hyponatremia.

Regarding post-race symptoms associated with race performance reported by finishers with EAH, the ultra-MTBer EAH-A-R2 reported muscle weakness. This symptom was frequent in all cycling races (R1,R2,R4). We assume that it could be related to higher race intensity during the races since EAH-A-R2 was also in the top finishers of the race and a more difficult racing terrain compared to the flat course in a 24-hour ultra-running event. Muscle weakness could be also associated with hypovolemia [52]. The myalgia reported in EAH-B-R3 and EAH-C-R4 may have been attributed to the extreme physical demands of the respective races, in all hyponatremic cases TTKG gradient increased and was > 10, presumably indicating an increased activity of aldosterone [2,53]. We assume that athletes suffered a great stress. The swelling and antidiuresis in EAH-B-R3 and EAH-C-R4 may have been a result of fluid overload, thus further investigation is warranted. The consensus on EAH states that it left untreated, symptoms of EAH can digress rapidly [48], in the current study however, reported symptoms were left untreated in the aftermath of the races. Nonetheless, no severe symptomatic case of EAH encephalopathy associated with dehydration has been reported in literature [52]. Subjects EAH-A-R2, EAH-B-R3 and EAH-C-R4 were contacted 24 h and 72 h after their races. Each reported that they all were fully oriented and without evidence of altered mental status and they did not require medical attention for hyponatremia. Since no hyponatremic athlete used NSAIDs we propose that NSAIDs did not influence an prevalence of EAH in the present subjects. Because both groups with and without EAH reported similar symptoms, no subject required medical attention for post-race hyponatremia, and no finisher had seizures or respiratory mistress during or within 24 h of the race finishing, we conclude that no hyponatremic finisher had EAH encephalopathy or pulmonary edema.

### Blood and urine parameters in hyponatremic finishers (n = 3)

Plasma volume increased in EAH-A-R2 and EAH-B-R3 and decreased in EAH-C-R4. Plasma osmolality remained stable and urine osmolality increased in all cases. In all hyponatremic cases (*i.e.* EAH-A-R2, EAH-B-R3 and EAH-C-R4) the transtubular potassium gradient increased and was > 10, presumably indicating an increased activity in aldosterone [54-56]. Previous work has suggested that EAH could promote rhabdomyolysis through changes in intracellular potassium or calcium concentrations [23]. Therefore, rhabdomyolysis could be a stimulus for EAH via the syndrome of SIADH mechanism [12,57] given the physiological demands of these races. EAH-A-R2 and EAH-B-R3 were hyperhydrated and EAH-C-R4 was dehydrated according to blood parameters. Hyponatremic cases EAH-A-R2 and EAH-B-R3 were dehydrated according to urinary parameters, however increased urinary sodium losses could be compatible with SIADH and they were overhydrated. Urine [Na^+^] decreased only in EAH-C-R4 possibly due to stimulation of the RAAS. The lower plasma [Na^+^] and the subsequent development of EAH in EAH-A-R2, EAH-B-R3 and EAH-C-R4 may be attributed to overdrinking, the retention of fluid because of inadequate suppression of vasopressin secretion, impaired mobilization of osmotically inactive sodium stores, and/or inappropriate inactivation of osmotically active sodium.

### Changes in plasma [Na^+^], plasma [K^+^], hematocrit, plasma volume, and plasma osmolality in normonatremic finishers (n = 50)

Plasma [Na^+^] remained stable with a non-significant decrease in all normonatremic finishers in the 24-hour races (R1-R3), but significantly decreased in the multi-stage race (R4). In the multi-stage race (R4) we must consider the possibility of interstitial swelling that does not dissipate between stages. Hematocrit was stable in R1, R2, R4, and decreased in R3, and was not related to fluid intake in either race. Furthermore, plasma [K^+^] decreased in R3, although plasma [Na^+^] did non-significantly decrease in this race. Plasma volume decreased in R1 and increased in R2, R3 and R4, and Δ plasma volume was not related to post-race plasma [Na^+^] or Δ plasma [Na^+^] in either race. The hemodilution seen in R2, R3 and R4 may have been a result of prolonged stress [23]. In longer ultra-endurance races plasma volume might increase, because ultra-athletes may retain a fluid reserve in the interstitial fluid of the extracellular fluid compartment [20,24], an increased plasma volume can be due to shift in body fluid. On the contrary, the reduction of plasma volume in R1 reflected in body mass reduction might be caused by dehydration, although the decreased plasma volume could be shown as a hemoconcentration due to the acute effect of strenuous endurance on hematological parameters [23]. The activation of the RAAS (renin-angiotensin-aldosterone-system) could lead to an enhanced retention of Na^+^ and free water, resulting in an increase in plasma volume and a decrease in plasma [Na^+^] [2,58]. Presumably, the increase in plasma volume in R2-R4 and the retention of water was due to an increased activity of both vasopressin and aldosterone [1,2,12,16,19,57,59].

Urinary indices are suggested as parameters of hydration status [53,60,61], however several studies have documented that they are not accurate measures of hydration status immediately following exercise activity [62] and plasma osmolality would be a better marker of hydration status in the situation of acute dehydration [58,63]. Plasma osmolality remained stable in all races with a non-significant increase despite a decrease in plasma [K^+^] in R3 and a decrease in plasma [Na^+^] in R4. An increase in transtubular potassium gradient could be responsible for a preservation of both plasma [Na^+^] and body water during ultra-endurance exercise due to an increased activity of aldosterone [8]. We assume that this may explain why plasma osmolality was stable in all races despite a loss in body mass. These findings support recent findings in Tam et al. [63] that the body primarily defends plasma [Na^+^] and aids at maintaining [Na^+^] and osmolality in plasma, but not body mass during endurance performance. In ultra-marathoners, plasma [Na^+^] and plasma osmolality are well regulated and do not change while drinking *ad libitum*[58].

### Changes in urine [Na^+^], urine [K^+^], urine specific gravity and urine osmolality in normonatremic finishers (n = 50)

Since hematological parameters such as plasma [Na^+^] or hematocrit were not valid indicators for the detection of mild hypohydration [61], urine parameters such as colour, urine specific gravity, and urine osmolality were considered to be valid indices of hydration status [61]. The decrease in body mass might be due to dehydration since urine specific gravity as a sign of dehydration [60,61] significantly increased in all cycling races (R1,R2,R4), and non-significantly increased in R3. Cyclists (R1,R2,R4) lost approximately 2.3% of body mass, with urine specific gravity of > 1.020 mg/l indicating dehydration [64], ultra-runners (R3) were minimally dehydrated according to changes in urine specific gravity.

On the contrary, the use of urine specific gravity as a marker of hydration status is time-dependent and shows only chronic dehydration, but not acute dehydration [53]. In ultra-endurance races, chronic dehydration seemed to result in a reduced renal function [8,55,56], because during long endurance performances renal blood flow and glomerular filtration rates decrease and limit the delivery of filtrate to the diluting segments of the kidney [55]. The increase in urine osmolality in all races (R1-R4) might be due to an increase in water permeability in the kidney, matching the fact that athletes urinated less frequently [2]. This could lead to impairments of free water excretion in R1, R2 and R4 with indicators of a more chronic than an acute dehydration. Post-race symptoms reported by finishers in all races indicated this hypothesis. Glomerular filtration race significantly decreased and urine osmolality increased and it seemed to be a result in a change in renal function.

### Arginine vasopressin secretion, aldosterone activity and the prevalence of EAH

SIADH is also considered as a potentional mechanism to develop EAH [39], because arginine vasopressin (AVP) regulates body’s retention of water. Changes in sodium and potassium concentrations and osmolality in plasma and urine are also indirect markers for the activity of aldosterone [2,4,16,19,45] and AVP-secretion [12,42,43,45,57,59]. Urine [K^+^] significantly increased in R1 and R4, and urine specific gravity was associated with post-race urine [K^+^] in R4. On the contrary, urine [K^+^] in R2 and R3 remained stable, and urine [Na^+^] significantly decreased in R2 and R3, although the K^+^/Na^+^ ratio in urine was < 1 only in R3.

The increased urinary [Na^+^] losses could be compatible with SIADH in R2 and R3. In all races, the transtubular potassium gradient increased and was > 10 in R1, R3 and R4, probably due to an increased aldosterone activity. This change in aldosterone is associated with a change in the K^+^/Na^+^-ratio in urine, a positive ratio suggests an increased aldosterone activity [16,18]. In all races (R1-R4), the K^+^/Na^+^-ratio in urine increased. The K^+^/Na^+^-ratio in urine was < 1.0 only in R3, suggesting that more potassium than sodium was excreted through the kidney, however the K^+^/Na^+^-ratio in urine was > 1 in R1, R2 and R4. Body water increase with simultaneous dehydration (R2-R4) might be possibly due to endocrine-induced renal water retention, in order to maintain the metabolic processes that are required for energy supply and blood flow during prolonged exercise [54].

Finishers were more hyperhydrated than dehydrated in R3. Apart from fluid overload, however, other mechanisms may have lead to water retention in R3, such as protein catabolism [54]. In a 24-hour running race, Fellmann et al. [59] found an increase in plasma volume, aldosterone and AVP. Stuempfle et al. [24] showed an increased activity of both aldosterone and AVP after an ultra-endurance race. Alternatively, there might be also an impairment in mobilization of osmotically-inactive sodium stores or inappropriate inactivation of osmotically-active sodium [11,18]. These cannot be determined from the present study.

### Fluid overload and the prevalence of EAH

Fluid overload is considered as the main risk factor for EAH [39,48]. We observed a higher than recommended fluid intake in EAH-A-R2. Fluid intake rates in EAH-B-R3 and EAH-C-R4 were at the upper limits of recommended fluid intakes. However, we did not observe the combination of overhydration and hyponatremia in the present work.

We expected that the prevalence of EAH would be higher in the 24-hour races (R1-R3) compared with the multi-stage MTB race (R4) due to the higher possibility of excessive drinking and their duration. This hypothesis was not supported in the present subjects since relative fluid consumption was similar in all groups (R1-R4) despite the different length of the races and the different weather conditions. Considering the aid stations and the nutrition provided, the races were comparable and only one ultra-MTBer in R1 and two MTBers in R4 used backpack type hydration packs. The average fluid intake in all races was 0.51 (0.1%) l/h which was in accordance with the International Marathon Medical Directors Association (IMMDA) [65] which recommends drinking *ad libitum* between 0.4 l/h and 0.8 l/h.

Fluid intake was the highest in R3 which had the coldest weather conditions and the highest prevalence of EAH (8.3%). In single stage ultra-distance races, Stuempfle et al. [24] reported a fluid overload caused by excessive fluid consumption during cold weather in a 161-km race in Alaska leading to both an increase in plasma volume and a decrease in plasma [Na^+^], although no athletes were classified as hyponatremic. Similar findings were also reported in 100-km ultra-marathoners [3] where the prevalence of EAH (4.8%) was in line with the findings of a study on 24-hour ultra-marathon runners [30]. Paradoxically, in R4 taking place in the warmest conditions, the finishers had the lowest fluid intake. In studies of multi-stage MTB races [21,22] fluid intake also did not exceed 0.75 l/h and was between 0.34 l/h and 0.55 l/h in the respective races. Although in both multi-stage races [21,22] no case of EAH was documented, we found one hyponatremic case in R4 (EAH-C-R4). In another study investigating 196 road cyclists in a 109-km cycling race one athlete developed hyponatremic encephalopathy despite a modest fluid intake [64].

Fluid intake was inversely related to post-race plasma [Na^+^] in R2, and also the highest number of overhydrated but normonatremic finishers from all races according to Noakes et al. [39] occurred in this race, although an average overall fluid intake was in line with IMMDA recommendations. Regarding these findings, fluid intake was probably responsible for hyperhydration in four normonatremic finishers in R2. This finding underlines the classic hypothesis of the pathogenesis of EAH as reported by Noakes at al. [39]. On the contrary, only one overhydrated normonatremic finisher occurred in R1 with no prevalence of EAH. In agreement with the findings of Knechtle et al. [3], there was a decrease in body mass, plasma [Na^+^] showed no changes, and Δ body mass was not related to fluid intake in R1. The hourly fluid intake of 0.49 l (0.30 l - 0.70 l) in R1 was not sufficient to prevent dehydration, but with regards to *ad libitum* fluid intake, body fluid homeostasis was maintained. Since fluid intake was not related to Δ plasma volume nor to Δ plasma [Na^+^] in R1, the effective homeostasis must result from the buffering effect of the exchangeable osmotically inactive body sodium stores [39]. Regardless of the modest fluid consumption in all groups (R1-R4), finishers in R2, R3 and R4 were more hyperhydrated than euhydrated, and factors other than fluid intake seemed responsible for fluid regulation in ultra-athletes, such as a hormonal regulation by aldosterone [2,19,21,24,57] and inappropriate levels of the hormone vasopressin [42,43] and the exchangeable osmotically inactive body sodium stores [39].

### Changes in body mass and prevalence in EAH

An important finding of this study was that of the three participants who were hyponatremic post-race, no finisher showed an increase in body mass. Both EAH-A-R2 and EAH-B-R3 were euhydrated, while EAH-C-R4 was dehydrated as defined by Noakes et al. [39]. Another observation from our study was that body mass decreased in all normonatremic ultra-endurance athletes (ultra-MTBers, ultra-runners and MTBers) in the 24-hour races (R1-R3), and in the multi-stage MTB race (R4). Δ body mass varied from a 6.6% loss in body mass to a 3.4% gain in body mass. EAH is more commonly associated with overhydration. In a recent study by Hoffman et al. [11], 18.5% of the finishers were dehydrated. Of those with EAH, 35.6% were euhydrated, and 35.6% were dehydrated. In 887 finishers of a 161-km ultramarathon, Δ body mass varied from an 8% loss to a 10% gain [11]. Top finishers in the ultra-MTBers (R1,R2) and the ultra-runners (R3) varied in Δ body mass from a 0.7% gain to a 6.6% loss and in the MTBers (R4) from a 3.4% gain in body mass to a 4.3% loss in body mass. On average, finishers in R1-R4 were euhydrated as defined by Noakes [39].

An extremely hot or cold environment is considered as a risk factor for EAH [12,40], however we found no relationship between the prevalence of EAH and the ambient temperature in the present study. The 24-hour MTB race (R1) was held in a warm weather with low humidity during the whole race and the multi-stage race (R4) was held in typical hot summer weather, however with higher humidity (Table 1). The 24-hour MTB race (R2) was in more variable weather conditions with some precipitations, higher temperature fluctuations and high humidity (Table 1). The 24-h running race (R3) was held in colder weather with heavy precipitations compared with races R1, R2 or R4. In a recent study with 887 observations of weight change in a 161-km running race, Hoffman et al. [11] found significant correlations for percentage Δ body mass and percentage of dehydrated runners with ambient temperature. In the 24-hour races R1 and R2 large differences in ambient temperature appeared during the day and night, which are similar to the changes seen by Lebus et al. in the ultra-runners in a 161-km ultra-marathon [7]. The lowest Δ body mass in R3 might be also due to a colder temperature than in other races, because of a wind chill and heavy raining during the race, there was probably less sweat loss. R1 and R4 were held in favorable weather conditions in contrast with the colder ambient temperatures in R2 and R3, moreover accompanied with rain during the whole race. The highest number of dehydrated athletes was in R4 (the multi-stage race), on the contrary, the least number of overhydrated finishers was in R1 (the 24-hour MTB race) with no case of EAH. Higher Δ body mass were seen in R1 and R4 compared to races held under colder conditions (R2,R3). Although there were large differences in ambient temperatures during the day and night, EAH did not occur in R1 in very high ambient temperature. Therefore we concluded that like in Hoffman et al. [11] and Knechtle et al. [15] the environmental conditions probably had an influence on race performance, but not on the prevalence of EAH in our subjects in these concrete races. The present work is also in agreement with previous studies [11,38] showing that while a greater ambient temperature was associated with the number of dehydrated finishers, it was not associated with a larger number of overhydrated finishers.

The hypothesis that body mass losses would have no influence on race performance [11] was supported in R2 (the 24-hour MTB race). Δ body mass was negatively related to race performance, finishers with the greatest body mass losses tended to have a better race performance such as a higher number of achieved kilometers. The significant relationship between percentage Δ body mass and race time showed that the fastest runners tended to lose more body mass as observed by Hoffman et al. [11] in a 161-km ultra-marathon and Kao et al. [32] in a 24-hour running race. Also, in Zouhal et al. [47] a loss in body mass did not affect performance, and in Knechtle et al. [15] faster runners in a 100-km ultra-marathon lost more body mass than slower runners. These data support the finding that Δ body mass during exercise may not reflect exact changes in hydration status [20,60], and a loss in body mass did not impair race performance. Presumably, the decrease in body mass in the present athletes in R2 could also be due to dehydration [60], or changes in body mass representing a balance of fluid and energy intake and fluid and energy losses from external and internal sources with significant fat mass losses during the race [26,37]. We assume that the loss in body mass could be also due to a substrate losses as well as fluid losses.

The additional finding that in any race post-race body mass or Δ body mass was negatively related to post-race plasma [Na^+^] warrants further investigation. These findings contrast with those from previous studies of other endurance events where post-race plasma [Na^+^] has been found to be negatively related to percentage Δ body mass [1,3,44,53]. Moreover, Δ body mass and % Δ body mass were positively related to post-race plasma [Na^+^] in ultra-runners (R3). Finishers with lower levels of plasma [Na^+^] had higher losses in body mass. A direct positive relationship between post-race plasma [Na^+^] and Δ body mass was reported by Hoffman et al. [11,38], Lebus et al. [7] and by Reid et al. [66], in contrast to what has been observed for many other races. Hoffman et al. [11] provided in the latest study the other side of the inverted-U curve to support the depletional model of EAH. Sodium losses, impairment in mobilization of osmotically inactive sodium stores and/or inappropriate inactivation of osmotically active sodium are alternative explanations. The relative importance of each of these factors cannot be determined from the present study.

### Race pace and prevalence of EAH

Despite other influences, a lower race pace could also increase the risk of EAH [39]. We hypothesized that the prevalence of EAH would be higher in ultra-runners in a 24-hour race, since they compete at a slower pace compared to ultra-cyclists in a 24-hour race. The important finding was that two (4.9%) of all 41 cyclists and one (8.3%) of 12 runners in our study developed EAH which was consistent with our premises. It should be taken into account that race speed and the number of achieved kilometers (*i.e.* race performance) during a 24-hour race might depend on physical condition, motivation, tactics or other factors [35,36,66].

The performance of the best athletes in a 24-hour MTB race was as fast at the end as at the beginning of a race, and the decrease or the increase in race speed has to do with tactics in the race, not overall pace [66]. It is difficult to compare race speed between cyclists and runners. However, the comparison of race performance of cases with EAH showed different results. In the 24-hour MTB races, EAH-A-R2 was a cyclist with a higher speed (18.4 km/h) and a better race performance (*i.e.* 9^th^ place from 116 participants in solo category) in comparison with the other finishers in R2 (Table 2). EAH-B-R3 was even the best in absolute ranking (*i.e.* 1^st^ place from 48 participants) with an average running speed of 9.2 km/h. Moreover, in R2 and R3, race performance was negatively associated with post-race plasma [Na^+^]. Finishers with lower post-race [Na^+^] in R2 and R3 achieved more kilometers during the 24 hours. These findings supported our results, where two of three hyponatremic athletes in our study were among the top finishers in our races. Presumably, the specific character of 24-hour races might explain this contradictory finding. The better performance seen in the faster runners is influenced by numerous reasons, such as the motivation to achieve a higher number of kilometers or better race time [35,36,66]. On the contrary, EAH-C-R4 was one of the slower cyclists with the absolute ranking 158^th^ place from 160 finishers, and with an average cycling speed of 6.5 km/h. Therefore, only in EAH-C-R4 we can assume that race speed was one of the factors which influenced EAH in our tested group.

### Fluid intake and race performance

An important finding was the fact that in the ultra-MTBers (R1,R2), fluid intake was positively related to the number of kilometers achieved during 24-hour MTB race, which is in agreement with previous studies [3,15,25,30,47]. The ultra-MTBers in the 24-hour MTB races R1 and R2 who drank more finished ahead of those who drank less. Furthermore, the ultra-MTBers in 24-hour MTB R2 with greater body mass losses achieved more kilometers in the race than those with lower body mass losses.

In a recent study, Knechtle et al. showed similar findings in 24-hour ultra-runners [30]. In contrast to the ultra-MTBers in R1 and R2, in the ultra-runners in R3 fluid intake was not related to race performance. We assume that the ultra-MTBers in R1 and R2 with a better race performance who did not develop EAH drank more than the others, however, still in accordance with IMMDA. In 219 runners in a 100-km ultra-marathon, the faster runners had a support crew to provide drinks in contrast to the slower runners with no support crew [15]. Presumably, also our faster ultra-MTBers used this possibility of an additional fluid intake. In Knechtle et al. [15], the faster athletes who probably had a higher sweating rate lost more fluids and consequently drank more fluids. The finding that fluid intake was positively correlated with race performance suggests that athletes in R1 and R2 were drinking appropriately. Faster athletes were working harder and required more water than slower athletes. We hypothesised that in cases of fluid overload, fluid intake would be related to post-race body mass, Δ body mass, post-race plasma [Na^+^], and Δ plasma [Na^+^], respectively. In none of the races was fluid intake associated with post-race body mass, Δ body mass, Δ plasma [Na^+^], Δ plasma volume, or Δ urine specific gravity.

Another finding was that the finishers with a better race performance had lower post-race plasma [Na^+^] in R2 and R3, and a higher body mass loss in R2. Also in Hoffman et al. [11], Knechtle et al. [15] and Noakes [63] faster runners tended to lose more body mass. Likewise, fluid intake was negatively associated with Δ body mass in a recent study [25]. In a 24-hour running race Δ body mass showed no association with post-race plasma [Na^+^], however, no subject developed EAH [31]. Moreover, fluid intake correlated negatively to average running speed [31].

However, it is difficult to explain the decrease in body mass despite the increased fluid intake and the lower post-race plasma [Na^+^]. In a recent study, faster runners lost more body mass, and faster runners drank more fluid than slower runners [65]. Also, faster ultra-MTBers in R2 lost more body mass although they drank more. We assume that the present ultra-athletes who ingested more fluid probably had a higher sweat loss during the race than the slower ones. The athletes who run the fastest will have the highest sweat rates. If they do not drink more than others they will finish with the greatest levels of body mass loss and hence the highest levels of dehydration [42]. In some instances fluid may have taken the place of food in terms of energy consumed. In a study of 2,135 endurance athletes including marathon runners and triathletes, plasma [Na^+^] decreased despite of an increased fluid intake, however body mass also decreased [39].

### Limitations

It was not possible in these races to determine urinary excretion of the finishers precisely since the athletes were not able to correctly record it during the race. Since ultra-endurance performance is associated with skeletal muscle damage [67], we have to investigate also the role of muscle damage in causing a decrease in skeletal muscle mass or fat mass.

## Conclusions

Overall prevalence of EAH was 5.7% and was not higher compared to existing reports for other ultra-endurance athletes competing in other countries. No ultra-MTBer developed EAH in the 24-hour MTB race (R1). One ultra-MTBer in the 24-hour MTB race (R2), one ultra-runner in the 24-hour running race (R3) and one MTBer in the multi-stage MTB race (R4) developed EAH with mild symptoms. To support the trend of the prevalence of EAH in the Czech Republic and to clarify the cause it is necessary to observe ultra-endurance athletes in a number of different races or a long time and repeatedly. The lower plasma sodium and the subsequent development of EAH may be attributed to overdrinking, a pituitary secretion of the hormone vasopressin, impaired mobilization of osmotically inactive sodium stores, and/or inappropriate inactivation of osmotically active sodium. Future studies need to investigate the change in body composition. A loss in body mass of >3% does not appear to adversely affect performance despite *ad libitum* fluid consumption being advised.

## Competing interests

The authors declare that they have no competing interests.

## Authors’ contributions

DCH and BK developed the objectives of the study and intervention, DCH managed recruitment and data collection, DCH and AZ participated in the practical measurement in all field studies, DCH and IT performed statistical analysis, DCH, BK and TR lead the drafting of the manuscript, interpreted the findings and critically reviewed the manuscript. All authors read and approved the final manuscript.
